# International Changes in COVID-19 Clinical Trajectories Across 315 Hospitals and 6 Countries: Retrospective Cohort Study

**DOI:** 10.2196/31400

**Published:** 2021-10-11

**Authors:** Griffin M Weber, Harrison G Zhang, Sehi L'Yi, Clara-Lea Bonzel, Chuan Hong, Paul Avillach, Alba Gutiérrez-Sacristán, Nathan P Palmer, Amelia Li Min Tan, Xuan Wang, William Yuan, Nils Gehlenborg, Anna Alloni, Danilo F Amendola, Antonio Bellasi, Riccardo Bellazzi, Michele Beraghi, Mauro Bucalo, Luca Chiovato, Kelly Cho, Arianna Dagliati, Hossein Estiri, Robert W Follett, Noelia García Barrio, David A Hanauer, Darren W Henderson, Yuk-Lam Ho, John H Holmes, Meghan R Hutch, Ramakanth Kavuluru, Katie Kirchoff, Jeffrey G Klann, Ashok K Krishnamurthy, Trang T Le, Molei Liu, Ne Hooi Will Loh, Sara Lozano-Zahonero, Yuan Luo, Sarah Maidlow, Adeline Makoudjou, Alberto Malovini, Marcelo Roberto Martins, Bertrand Moal, Michele Morris, Danielle L Mowery, Shawn N Murphy, Antoine Neuraz, Kee Yuan Ngiam, Marina P Okoshi, Gilbert S Omenn, Lav P Patel, Miguel Pedrera Jiménez, Robson A Prudente, Malarkodi Jebathilagam Samayamuthu, Fernando J Sanz Vidorreta, Emily R Schriver, Petra Schubert, Pablo Serrano Balazote, Byorn WL Tan, Suzana E Tanni, Valentina Tibollo, Shyam Visweswaran, Kavishwar B Wagholikar, Zongqi Xia, Daniela Zöller, The Consortium For Clinical Characterization Of COVID-19 By EHR (4CE), Isaac S Kohane, Tianxi Cai, Andrew M South, Gabriel A Brat

**Affiliations:** 1 Department of Biomedical Informatics Harvard Medical School Boston, MA United States; 2 BIOMERIS (BIOMedical Research Informatics Solutions) Pavia Italy; 3 Clinical Research Unit Botucatu Medical School São Paulo State University Botucatu Brazil; 4 Division of Nephrology Department of Medicine Ente Ospedaliero Cantonale Lugano Switzerland; 5 Department of Electrical, Computer and Biomedical Engineering University of Pavia Pavia Italy; 6 Information Technology Department Azienda Socio-Sanitaria Territoriale di Pavia Pavia Italy; 7 Unit of Internal Medicine and Endocrinology Istituti Clinici Scientifici Maugeri SpA SB IRCCS Pavia Italy; 8 Massachusetts Veterans Epidemiology Research and Information Center Veterans Affairs Boston Healthcare System Boston, MA United States; 9 Department of Electrical Computer and Biomedical Engineering University of Pavia Pavia Italy; 10 Department of Medicine Massachusetts General Hospital Boston, MA United States; 11 Department of Medicine David Geffen School of Medicine University of California, Los Angeles Los Angeles, CA United States; 12 Health Informatics Hospital Universitario 12 de Octubre Madrid Spain; 13 Department of Learning Health Sciences University of Michigan Medical School Ann Arbor, MI United States; 14 Department of Biomedical Informatics University of Kentucky Lexington, KY United States; 15 Department of Biostatistics, Epidemiology, and Informatics University of Pennsylvania Perelman School of Medicine Philadelphia, PA United States; 16 Institute for Biomedical Informatics University of Pennsylvania Perelman School of Medicine Philadelphia, PA United States; 17 Department of Preventive Medicine Northwestern University Chicago, IL United States; 18 Institute for Biomedical Informatics University of Kentucky Lexington, KY United States; 19 Medical University of South Carolina Charleston, SC United States; 20 Department of Computer Science Renaissance Computing Institute University of North Carolina at Chapel Hill Chapel Hill, NC United States; 21 Department of Biostatistics Harvard T.H. Chan School of Public Health Boston, MA United States; 22 Department of Anaesthesia National University Health System Singapore Singapore; 23 Institute of Medical Biometry and Statistics Faculty of Medicine and Medical Center University of Freiburg Freiburg Germany; 24 Michigan Institute for Clinical & Health Research Informatics University of Michigan Ann Arbor, MI United States; 25 Laboratory of Informatics and Systems Engineering for Clinical Research Istituti Clinici Scientifici Maugeri SpA SB IRCCS Pavia Italy; 26 Clinical Hospital of Botucatu Medical School São Paulo State University Botucatu Brazil; 27 Informatique et archivistique médicales unit Bordeaux University Hospital Bordeaux France; 28 Department of Biomedical Informatics University of Pittsburgh Pittsburgh, PA United States; 29 Department of Neurology Massachusetts General Hospital Boston, MA United States; 30 Department of Biomedical Informatics Hôpital Necker-Enfants Malade, Assistance Publique Hôpitaux de Paris University of Paris Paris France; 31 Department of Biomedical Informatics, Institute for Digital Medicine National University Health System Singapore Singapore; 32 Internal Medicine Department Botucatu Medical School São Paulo State University Botucatu Brazil; 33 Department of Computational Medicine & Bioinformatics, Internal Medicine, Human Genetics, and Public Health University of Michigan Ann Arbor, MI United States; 34 Division of Medical Informatics Department of Internal Medicine University of Kansas Medical Center Kansas City, KS United States; 35 Data Analytics Center University of Pennsylvania Health System Philadelphia, PA United States; 36 Department of Medicine National University Health System Singapore Singapore; 37 Department of Neurology University of Pittsburgh Pittsburgh, PA United States; 38 Section of Nephrology Department of Pediatrics Brenner Children's Hospital, Wake Forest School of Medicine Winston Salem, NC United States; 39 see Authors’ Contributions

**Keywords:** SARS-CoV-2, electronic health records, federated study, retrospective cohort study, meta-analysis, COVID-19, severe COVID-19, laboratory trajectory

## Abstract

**Background:**

Many countries have experienced 2 predominant waves of COVID-19–related hospitalizations. Comparing the clinical trajectories of patients hospitalized in separate waves of the pandemic enables further understanding of the evolving epidemiology, pathophysiology, and health care dynamics of the COVID-19 pandemic.

**Objective:**

In this retrospective cohort study, we analyzed electronic health record (EHR) data from patients with SARS-CoV-2 infections hospitalized in participating health care systems representing 315 hospitals across 6 countries. We compared hospitalization rates, severe COVID-19 risk, and mean laboratory values between patients hospitalized during the first and second waves of the pandemic.

**Methods:**

Using a federated approach, each participating health care system extracted patient-level clinical data on their first and second wave cohorts and submitted aggregated data to the central site. Data quality control steps were adopted at the central site to correct for implausible values and harmonize units. Statistical analyses were performed by computing individual health care system effect sizes and synthesizing these using random effect meta-analyses to account for heterogeneity. We focused the laboratory analysis on C-reactive protein (CRP), ferritin, fibrinogen, procalcitonin, D-dimer, and creatinine based on their reported associations with severe COVID-19.

**Results:**

Data were available for 79,613 patients, of which 32,467 were hospitalized in the first wave and 47,146 in the second wave. The prevalence of male patients and patients aged 50 to 69 years decreased significantly between the first and second waves. Patients hospitalized in the second wave had a 9.9% reduction in the risk of severe COVID-19 compared to patients hospitalized in the first wave (95% CI 8.5%-11.3%). Demographic subgroup analyses indicated that patients aged 26 to 49 years and 50 to 69 years; male and female patients; and black patients had significantly lower risk for severe disease in the second wave than in the first wave. At admission, the mean values of CRP were significantly lower in the second wave than in the first wave. On the seventh hospital day, the mean values of CRP, ferritin, fibrinogen, and procalcitonin were significantly lower in the second wave than in the first wave. In general, countries exhibited variable changes in laboratory testing rates from the first to the second wave. At admission, there was a significantly higher testing rate for D-dimer in France, Germany, and Spain.

**Conclusions:**

Patients hospitalized in the second wave were at significantly lower risk for severe COVID-19. This corresponded to mean laboratory values in the second wave that were more likely to be in typical physiological ranges on the seventh hospital day compared to the first wave. Our federated approach demonstrated the feasibility and power of harmonizing heterogeneous EHR data from multiple international health care systems to rapidly conduct large-scale studies to characterize how COVID-19 clinical trajectories evolve.

## Introduction

From January 2020 to June 2021, the COVID-19 pandemic has resulted in over 170 million confirmed cases of SARS-CoV-2 infection and 3.7 million confirmed deaths worldwide [1]. Similar to previous viral pandemics, the resurgence in SARS-CoV-2 infections and subsequent hospitalizations since the first documented outbreaks have been characterized by a series of “waves.” To date, there have been reports of at least two waves in numerous countries, including an initial one in the Spring of 2020 and a resurgence of cases in the Summer and Fall of 2020 [1-10]. A limited number of single-center studies have reported differences in laboratory values, demographic composition, and disease management between patients with COVID-19 admitted in the first and second waves [6,8,11,12]. Thus, there is substantial interest in comparing the clinical trajectories of patients with SARS-CoV-2 who were hospitalized across different waves of the pandemic to better understand the rapidly evolving epidemiology, pathophysiology, and health care dynamics of the COVID-19 pandemic. This may further inform health care workers, policymakers, and public health experts on how to anticipate potential additional waves due to SARS-CoV-2 variants [13].

Single-center studies are limited in scope, power, and generalizability, and there is a need for robust multicenter analyses using multinational cohorts that compare first and second wave patient characteristics. The goal of this study was to use a federated electronic health record (EHR)-based approach to examine international temporal trends in the clinical trajectories of patients hospitalized with SARS-CoV-2 across 6 countries obtained from contributing health care systems in the Consortium for Clinical Characterization of COVID-19 by EHR (4CE) [14], an international research collaborative of more than 300 hospitals across 7 countries that collects patient-level EHR data to study the epidemiology and clinical course of COVID-19. We collected data from 26 participating international health care systems covering 79,613 hospitalized patients with SARS-CoV-2 to study changes in (1) hospitalization rates across calendar time; (2) risk of developing severe COVID-19; and (3) mean laboratory values and laboratory testing rates between the first and second waves. We stratified severity risk analyses by country and demographic subgroups.

## Methods

### Description of the Federated Approach: Participating Health Care Systems, Local Data Collection, and Central Data Aggregation

**Figure 1:**
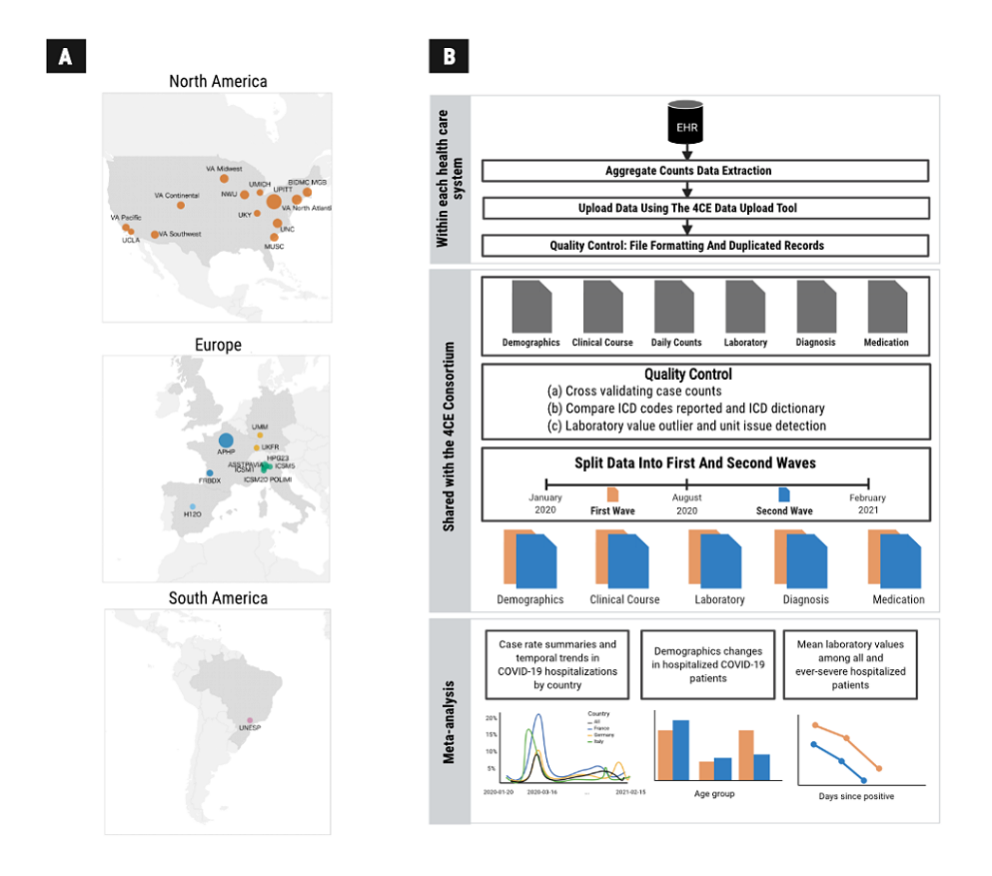
(A) World map with health care systems. (B) Schematic of the federated electronic health record (EHR)-based study involving health care systems from 6 countries. 4CE: Consortium for Clinical Characterization of COVID-19 by EHR; ICD: International Classification of Diseases.

Our analyses were performed on EHR data collected from 315 hospitals (affiliated with 26 regional health care systems) across the following 6 countries: Brazil, France, Germany, Italy, Spain, and the United States [14,15]. In the United States, we grouped the 170 Veterans Affairs (VA) hospitals into 5 regional health care systems [16]. See Table 1 for details about participating health care systems and Figure 1 for a map of participating health care systems.

**Table 1:** Participating health care systems, metadata on the number of hospitals and beds, and hospitalization date used to define the first and second wave cohorts.

Health care system	Country	Hospitals, n	Beds, n	Inpatient discharges/year, n	First wave date range	Second wave date range	First wave sample size, n	Second wave sample size, n
Assistance Publique - Hôpitaux de Paris	France	39	20,098	1,375,538	January 29, 2020, to August 10, 2020	August 11, 2020, to November 06, 2020	9827	4584
Azienda Socio-Sanitaria Territoriale della provincia di Pavia	Italy	7	958	29,103	February 28, 2020, to April 30, 2020	May 01, 2020, to February 15, 2021	945	1543
Beth Israel Deaconess Medical Center	United States	1	673	40,752	March 23, 2020, to September 02, 2020	September 03, 2020, to February 22, 2021	685	585
Bordeaux University Hospital	France	3	2676	130,033	January 23, 2020, to July 31, 2020	August 01, 2020, to November 07, 2020	331	439
Hospital Universitario 12 de Octubre	Spain	1	1256	45,035	March 01, 2020, to July 20, 2020	July 21, 2020, to February 28, 2021	2369	3730
Azienda Socio-Sanitaria Territoriale Papa Giovanni XXIII Bergamo	Italy	1	1080	45,000	February 25, 2020, to May 21, 2020	May 22, 2020, to November 13, 2020	1533	371
Istituto Clinico Scientifico Maugeri Pavia Hospital	Italy	1	426	8616	February 29, 2020, to May 08 2020	May 09, 2020, to November 16, 2020	113	58
Istituto Clinico Scientifico Maugeri Milano Hospital	Italy	1	200	2432	February 21, 2020, to May 08, 2020	May 09, 2020, to November 16, 2020	38	119
Istituto Clinico Scientifico Maugeri Lumezzane/Brescia Hospitals	Italy	1	149	1296	March 11, 2020, to May 08, 2020	May 09, 2020, to November 16, 2020	111	21
Mass General Brigham (Partners Healthcare)	United States	10	3418	163,521	March 11, 2020, to July 31, 2020	August 01, 2020, to February 28, 2021	2736	1735
Medical University of South Carolina	United States	8	1600	55,664	March 12, 2020, to May 25, 2020	May 26, 2020, to November 15, 2020	127	1482
Northwestern University	United States	10	2234	103,279	March 05, 2020, to July 31, 2020	August 01, 2020, to December 31, 2020	2313	3567
Policlinico di Milano	Italy	1	900	40,000	February 25, 2020, to August 01, 2020	August 02, 2020, to November 13, 2020	612	304
Medical Center, University of Freiburg	Germany	1	1660	71,500	March 13, 2020, to July 31, 2020	August 01, 2020, to February 28, 2021	186	490
University of California, LA	United States	2	786	40,526	March 10, 2020, to August 03, 2020	August 04, 2020, to November 13, 2020	425	151
University of Kentucky	United States	3	881	45,714	March 18, 2020, to July 07, 2020	July 08, 2020, to November 06, 2020	113	352
University of Michigan	United States	3	1000	49,008	March 09, 2020, to July 31, 2020	August 01, 2020, to February 28, 2021	745	1619
University Medicine Mannheim	Germany	1	1352	50,748	March 18, 2020, to August 03, 2020	August 04, 2020, to January 23, 2021	81	497
University of North Carolina at Chapel Hill	United States	11	3095	52,000	March 14, 2020, to June 05, 2020	June 06, 2020, to October 30, 2020	458	1525
Universidade Estadual Julio de Mesquita Filho	Brazil	1	490	28,167	April 01, 2020, to July 31, 2020	August 01, 2020, to February 28, 2021	171	425
University of Pittsburgh	United States	39	8085	369,300	March 13, 2020, to July 31, 2020	August 01, 2020, to February 28, 2021	685	5021
Veteran Affairs North Atlantic	United States	49	3594	151,075	March 01, 2020, to July 31, 2020	August 01, 2020, to February 04, 2021	1949	2984
Veteran Affairs Southwest	United States	29	3115	156,315	March 01, 2020, to July 31, 2020	August 01, 2020, to February 04, 2021	1679	4071
Veteran Affairs Midwest	United States	39	2686	145,468	March 01, 2020, to July 31, 2020	August 01, 2020, to February 04, 2021	1544	4617
Veteran Affairs Continental	United States	24	2110	113,260	March 01, 2020, to July 31, 2020	August 01, 2020, to February 04, 2021	1497	3495
Veteran Affairs Pacific	United States	29	2296	114,569	March 01, 2020, to July 31, 2020	August 01, 2020, to February 04, 2021	1194	3361
Total	N/A^a^	315	66,818	3,427,919	N/A	N/A	32,467	47,146

^a^N/A: not applicable.

Similar to our previous 4CE studies, we distributed a SQL database script to each of the contributing health care systems, which they ran on their patient-level EHR data to generate aggregate counts and statistics about their patient cohorts after gaining institutional review board approval [14,15,17]. Health care systems then uploaded their aggregate data via a central 4CE data upload tool. Aggregate data included hospital admission summaries over calendar time; dates of positive SARS-CoV-2 reverse transcription polymerase chain reaction tests; demographic counts for age, sex, and race groups; and daily trajectories of laboratory test values. Race data were only reported in participating health care systems from the United States and only included categories for black and white patients given the considerable heterogeneity present in race coding systems across health care system EHRs. A schematic of our workflow is presented in Figure 1, and further details of collected data are reported in Multimedia Appendix 1.

In order to ensure high-quality EHR data across health care systems and countries, extensive data quality control was performed. When sites uploaded the data using the 4CE data upload tool, an initial online quality control verified that all comma separated value (CSV) files were under the standard format, which included verification of the file and column names, column orders, data types, code values and ranges, and the absence of duplicated records. This step was crucial in ensuring proper downstream statistical analysis. At the central site, additional quality control steps were completed on all submitted data. These steps included cross-validating the consistency of the total case counts, checking that there are no negative values in patient counts, and verifying that no data types were missing. We also checked for consistency between the 3-digit International Classification of Diseases (ICD) codes and the ICD dictionary. To assess the general consistency of the laboratory data from each site with data observed from all health care systems and to detect outliers, we plotted laboratory values across time with all sites overlaid on each other. Using these plots, we were also able to check if certain laboratory values from specific health care systems were systematically lower or higher than for other sites, which usually indicated unit errors. If a health care system presented any quality control issues, the central site directly contacted its corresponding informaticians to resolve them.

### Cohort Identification

Our study included all patients hospitalized up to February 28, 2021, at participating 4CE health care systems with an admission date between 7 days before to 14 days after the date of the first positive SARS-CoV-2 reverse transcription polymerase chain reaction test result. We chose this time window in an effort to mitigate selection bias by identifying hospitalized patients who may have tested positive for SARS-CoV-2 before or after being admitted to a hospital. We further defined the first admission date within this −7 to +14-day time window as the index date, and “days since admission” and “hospital day” are referenced to this index date. EHR data were available for 79,613 patients.

We partitioned patients into first- and second-wave cohorts according to their index date. Although different regions had slightly varying temporal trajectories in COVID-19–related hospitalizations, our data indicated 2 predominant waves of hospitalizations, which we used to partition patients as follows: a first wave from January 1 to July 31, 2020, and a second wave from August 1, 2020, to February 28, 2021. Relatively few admissions occurred between July and August 2020 across all health care systems. Table 1 defines health care system-specific hospitalization date criteria for the first and second waves.

We further categorized patients as “ever-severe” using the validated 4CE COVID-19 severity algorithm that allows us to determine whether patients, at any time during their hospitalization, progressed to severe disease, regardless of their recovery from COVID-19 [18]. The algorithm leverages a set of EHR data elements to define severe COVID-19 including (1) laboratory tests for partial pressure of carbon dioxide or partial pressure of oxygen; (2) ordered medications for sedatives and anesthetics; (3) diagnosis codes for acute respiratory distress syndrome or ventilator-associated pneumonia; and (4) procedures such as endotracheal tube insertion and invasive mechanical ventilation [18].

### Statistical Analysis

Centralized random effect meta-analyses were performed to summarize individual health care system effect sizes. To account for heterogeneity between health care systems, we harmonized effect sizes using DerSimonian and Laird random effect meta-analysis [19]. Weights assigned to health care system effect sizes during meta-analysis were kept constant between corresponding first and second cohort analyses to facilitate effective comparisons between waves. All statistical analyses were performed using R software version 4.0.2 (R Foundation for Statistical Computing).

We estimated the intensity rate of hospitalizations over time within each participating health care system and averaged at the country level. Within each health care system, the intensity rate for a given calendar date was estimated as the proportion of patients in the cohort who were hospitalized on that date. We further summarized the prevalence of demographic subgroups in the first and second waves. We excluded the VA health care systems only when estimating the prevalence of demographic subgroups in our cohort due to their unique demographic profiles [20]. We report the prevalence of demographic subgroups including the VA health care systems in Multimedia Appendix 2.

We then estimated the absolute risk of severe COVID-19 in the first and second waves and the relative risk (RR) of severe COVID-19 in the second wave compared with the first wave. Within each health care system and over a set time period of interest, the absolute risk was estimated as the proportion of patients who ever developed severe disease among all patients in the corresponding cohort. We stratified these analyses by country and demographic subgroups. Analyses of absolute risk and RR for severe COVID-19 included all participating health care systems.

We then compared standardized mean laboratory test values stratified by disease severity at days 0, 1, and 7 to investigate changes in laboratory trajectories between the 2 waves. We focused on the following 6 blood laboratory values associated with worse outcomes and severe disease in patients with COVID-19: C-reactive protein (CRP), ferritin, fibrinogen, procalcitonin, D-dimer, and creatinine [21-28]. To facilitate effective comparisons, we defined standardized laboratory values as relative to each laboratory test’s harmonized value on the index date in the first wave. We also summarized the proportion of all and ever-severe patients having each laboratory test at days 0 to 14 stratified by country to examine any changes in clinical practice regarding laboratory testing.

### Ethics Approval

All study sites were responsible for and obtained ethics approval, as needed, from the appropriate ethics committee at their institutions.

### Data Sharing Statement

Deidentified aggregate data were provided by sites for this study. The 4CE provides samples of deidentified data collected by the consortium and some corresponding visualizations on the consortium website [29].

## Results

### Characteristics of the Study Population and Trends in Hospitalization

**Figure 2:**
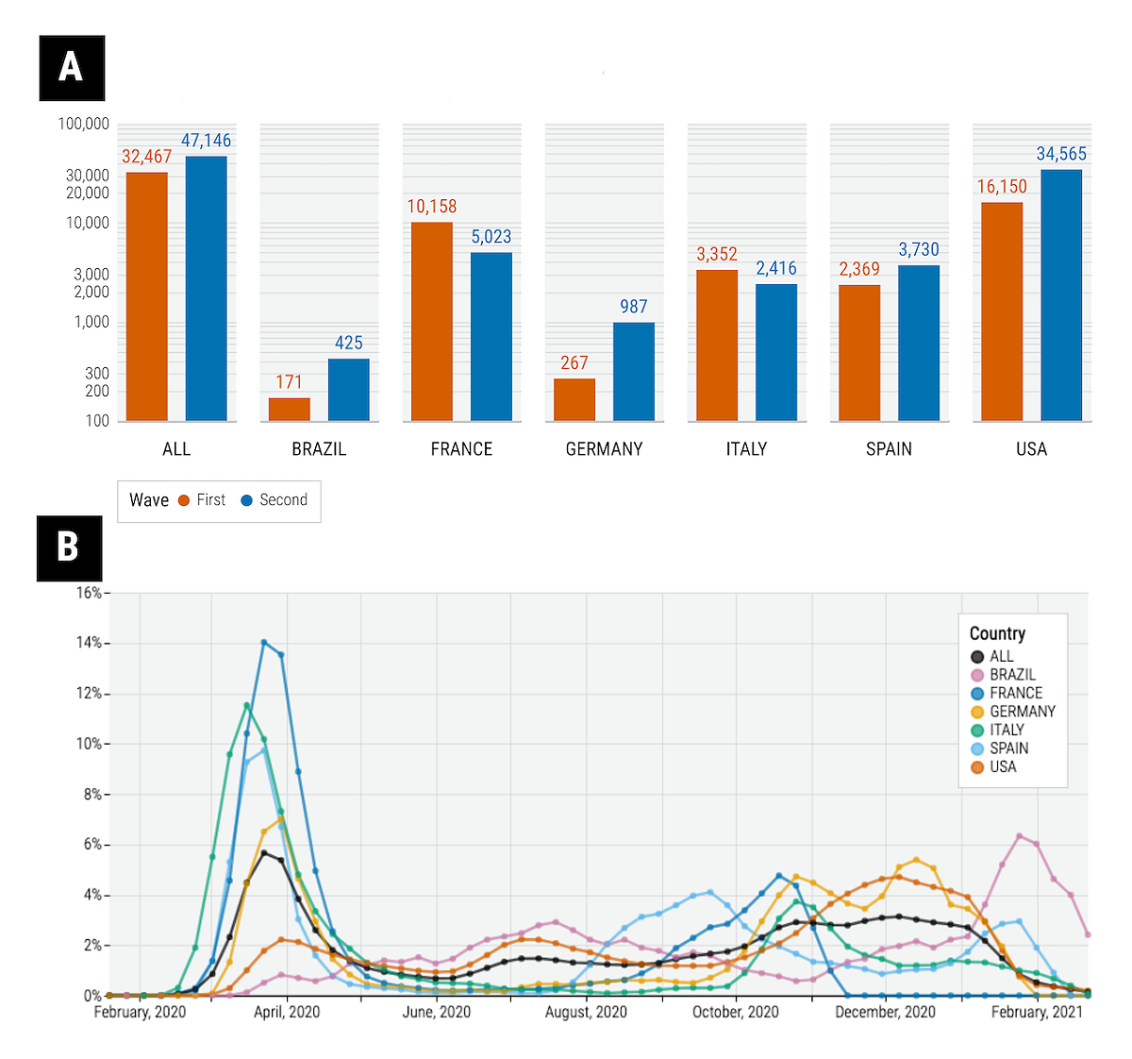
(A) Total hospitalizations in the cohort between the first and second waves. (B) The intensity rate of hospitalizations over time by country.

In the study population of 79,613 hospitalized patients with SARS-CoV-2, 32,467 were hospitalized during the first wave and 47,146 were hospitalized during the second wave. In this cohort, the United States represented the country with the most hospitalizations. As seen in Figure 2B, hospitalization rates generally peaked in March-April of 2020 and again in the final months of 2020 across all 6 countries.

**Figure 3:**
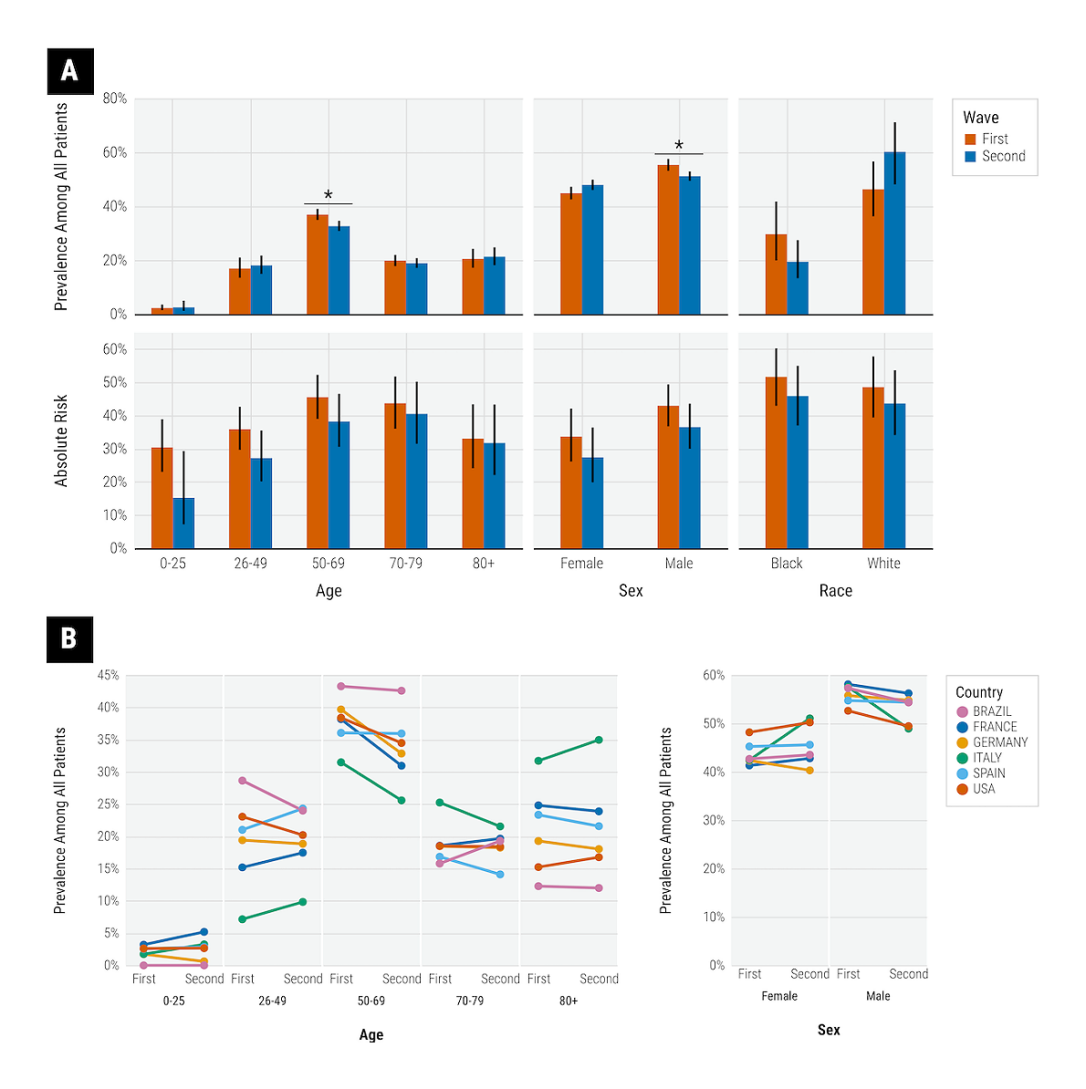
(A) Prevalence of demographic subgroups in the first and second waves for the entire cohort and estimated absolute risk for severe COVID-19 by demographic subgroup. (B) Country-level demographics of all patients by wave. Error bars indicate 95% CIs. *Significant difference by nonoverlapping 95% CIs.

We report the prevalence of demographic subgroups in Figure 3. Overall, there was a higher prevalence of male and older age patients in both waves. The prevalence of patients aged 50 to 69 years decreased significantly from the first wave (37.1%, 95% CI 35.0%-39.3%) to the second wave (32.3%, 95% CI 30.5%-34.2%). The prevalence of male patients also decreased from the first wave (55.3%, 95% CI 53.1%-57.5%) to the second wave (50.9%, 95% CI 49.1%-52.6%). There were no statistically significant changes in the prevalence of other age or sex groups for the entire cohort. At the country level, we observed that in Spain, the prevalence of patients aged 26 to 49 years increased significantly from 21.0% (95% CI 19.4%-22.7%) in the first wave to 24.3% (95% CI 23.0%-25.7%) in the second wave, while the prevalence of patients aged 70 to 79 years decreased significantly from 16.8% (95% CI 15.4%-18.4%) in the first wave to 14.1% (95% CI 13.0%-15.3%) in the second wave. In the United States, the prevalence of white patients increased (first wave: 46.3%, 95% CI 36.3%-56.6% vs second wave: 60.2%, 95% CI 48.1%-71.1%), while the prevalence of black patients decreased (first wave: 29.7%, 95% CI 20.0%-41.8% vs second wave: 19.4%, 95% CI 13.3%-27.5%), although both results did not reach statistical significance.

### Risk of Severe Disease in the First and Second Waves

**Figure 4:**
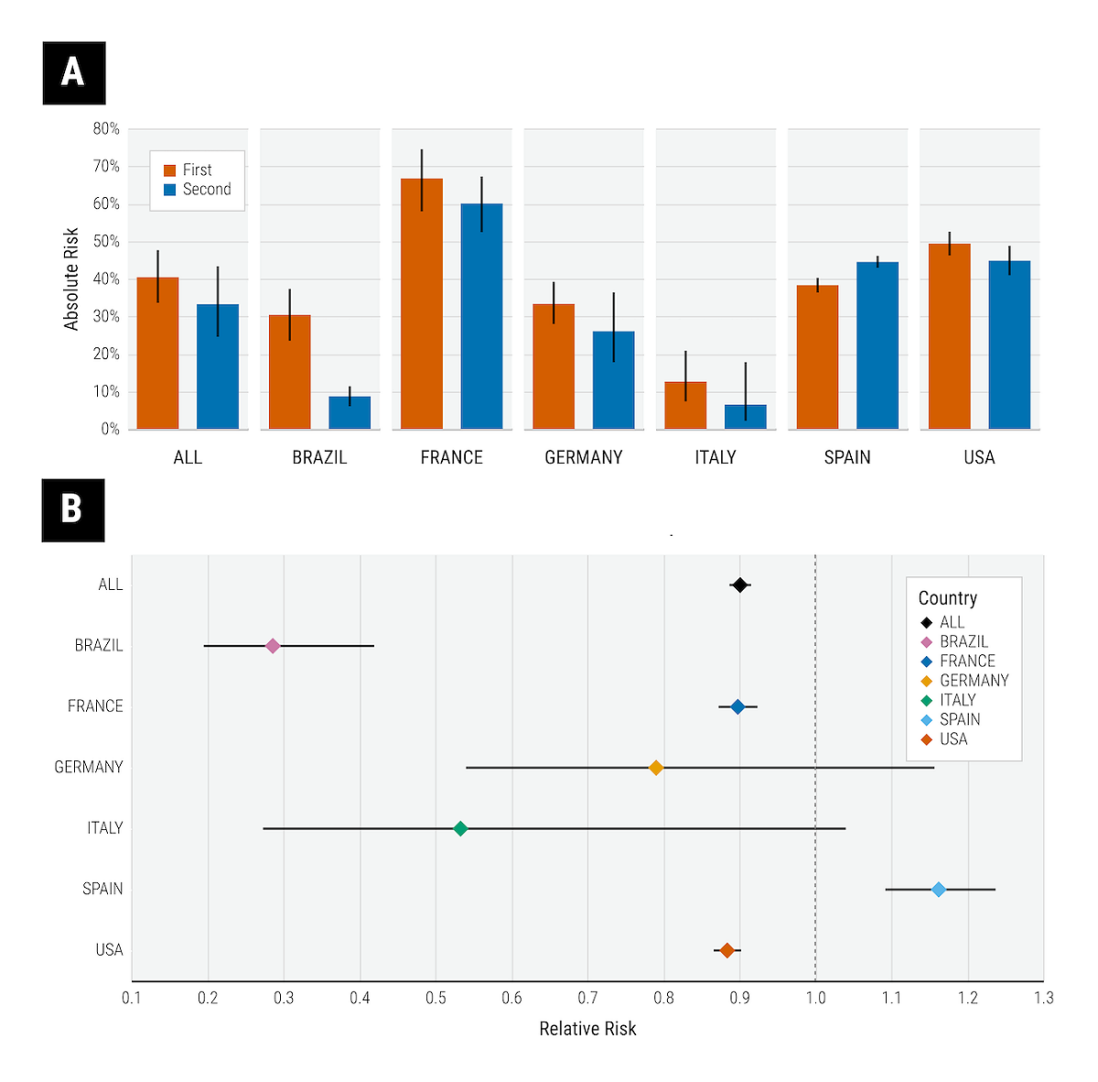
(A) Absolute risk for severe COVID-19 in the first and second waves. (B) Relative risk of severe COVID-19 in the second compared to the first wave stratified by country. Error bars indicate 95% CIs.

We report the absolute risk and RR for severe COVID-19 stratified by country in Figure 4. The absolute risk of severe disease was 0.40 (95% CI 0.34-0.48) in the first wave and 0.33 (95% CI 0.25-0.43) in the second wave. The absolute risk for severe disease varied significantly across countries in both waves (first wave vs second wave) as follows: Brazil, 30.1% vs 8.7%; France, 66.7% vs 60.1%; Germany, 33.3% vs 25.8%; Italy, 12.6% vs 6.5%; Spain, 38.3% vs 44.5%; United States, 49.4% vs 44.8%. The RR of severe disease in the second wave compared to the first wave was more comparable across Brazil (RR 0.29, 95% CI 0.20-0.42), France (RR 0.90, 95% CI 0.87-0.92), and the United States (RR 0.88, 95% CI 0.87-0.90). The observed RR was 0.78 (95% CI 0.52-1.17) in Germany and 0.53 (95% CI 0.27-1.04) in Italy, but the reduction in risk was not statistically significant. In contrast, patients in Spain (RR 1.16, 95% CI 1.09-1.24) had a slightly higher risk of severe COVID-19 in the second wave than in the first wave.

We report the absolute risk and RR for severe COVID-19 stratified by demographic subgroups in Figure 3A and Table 2, respectively. Across demographic subgroups in the second wave, there were significant reductions in risk among patients aged 26 to 49 years and 50 to 69 years; male and female patients; and black patients. RR effect sizes were generally comparable between sexes and between races. The reduction in risk in the second wave was slightly greater for younger age groups than for older age groups.

**Table 2:** Relative risk of severe disease in the second wave compared to the first wave stratified by demographic subgroups and by country.

Demographic group			All countries, RR^a^ (95% CI)		Brazil, RR (95% CI)		France, RR (95% CI)		Germany, RR (95% CI)		Italy, RR (95% CI)		Spain, RR (95% CI)		United States, RR (95% CI)
**Age (years)**															
	0-25	0.75 (0.56-1.02)		N/A^b^		1.50 (1.08-2.09)^c^		N/A		N/A		N/A		0.80 (0.59-1.09)	
	26-49	0.77 (0.63-0.94)^c^		0.31 (0.16-0.61)^c^		0.86 (0.57-1.31)		1.1 (0.51-2.45)		0.24 (0.07-0.78)^c^		1.08 (0.90-1.29)		0.81 (0.69-0.94)^c^	
	50-69	0.84 (0.72-0.97)^c^		0.23 (0.12-0.43)^c^		0.95 (0.89-1.01)		0.88 (0.56-1.39)		0.50 (0.17-1.46)		1.12 (1.02-1.23)^c^		0.88 (0.79-0.98)^c^	
	70-79	0.91 (0.80-1.02)		0.26 (0.12-0.60)^c^		1.00 (0.95-1.06)		0.84 (0.53-1.34)		1.38 (0.97-1.97)		1.16 (1.03-1.32)^c^		0.87 (0.76-0.99)^c^	
	≥80	1.01 (0.87-1.17)		0.62 (0.11-3.34)		0.93 (0.77-1.12)		0.87 (0.37-1.96)		1.13 (0.56-2.28)		1.49 (1.31-1.69)^c^		0.97 (0.83-1.15)	
**Sex**															
	Female	0.84 (0.73-0.96)^c^		0.22 (0.12-0.42)^c^		0.88 (0.84-0.92)^c^		0.59 (0.13-2.71)		0.87 (0.49-1.53)		1.13 (1.02-1.25)^c^		0.86 (0.76-0.98)^c^	
	Male	0.85 (0.76-0.95)^c^		0.32 (0.19-0.53)		0.93 (0.90-0.96)^c^		0.58 (0.25-1.34)		0.61 (0.29-1.25)		1.18 (1.10-1.28)^c^		0.89 (0.81-0.98)*^c^	
**Race**															
	Black	0.89 (0.81-0.98)^c^		N/A		N/A		N/A		N/A		N/A		0.89 (0.81-0.98)^c^	
	White	0.91 (0.80-1.03)		N/A		N/A		N/A		N/A		N/A		0.91 (0.80-1.03)	

^a^RR: relative risk.

^b^N/A: not applicable; no patients reported in specific demographic subgroups for certain countries.

^c^Statistically significant.

### Change in Mean Laboratory Values and Laboratory Testing Rates

**Figure 5:**
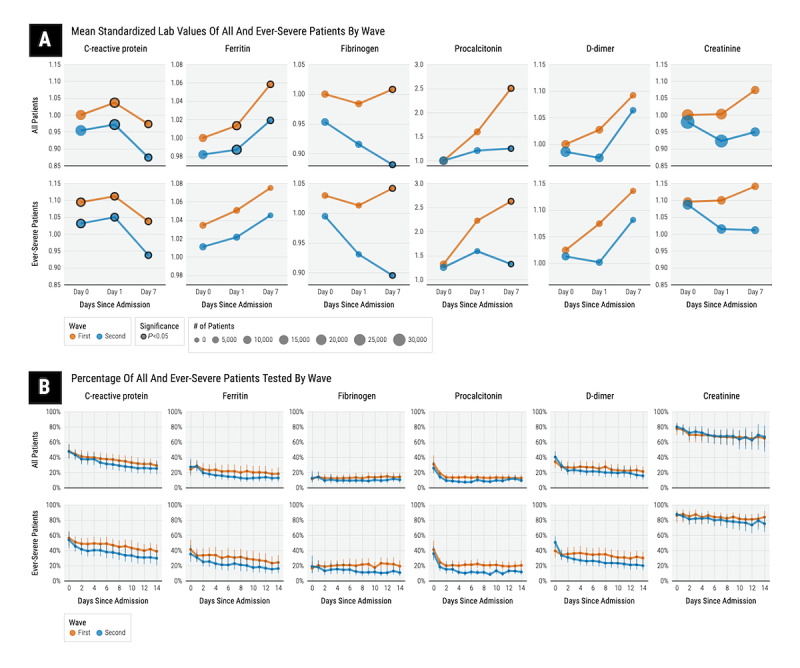
Standardized mean laboratory values (A) and corresponding laboratory testing rates (B) among all patients and those with severe disease in the first and second waves. Error bars indicate 95% CIs.

We report standardized mean laboratory values in the first and the second waves at days 0, 1, and 7 since the index date of admission for CRP, ferritin, fibrinogen, procalcitonin, D-dimer, and creatinine in Figure 5. Among all patients, we observed significantly lower mean CRP values throughout the first week of hospitalization on days 0, 1, and 7 in the second wave than in the first wave. All other mean laboratory values on day 0 were not significantly different between the first and second waves. At day 7, we further observed that the mean values of ferritin, fibrinogen, and procalcitonin were all significantly lower in the second wave than in the first wave.

Among patients with severe disease, we observed similar results with regard to comparing mean laboratory values between the first and second waves. Mean CRP values during the first week of hospitalization on days 0, 1, and 7 were significantly lower in the second wave than in the first wave. Mean fibrinogen and procalcitonin values were significantly lower on day 7 in the second wave than in the first wave.

**Figure 6:**
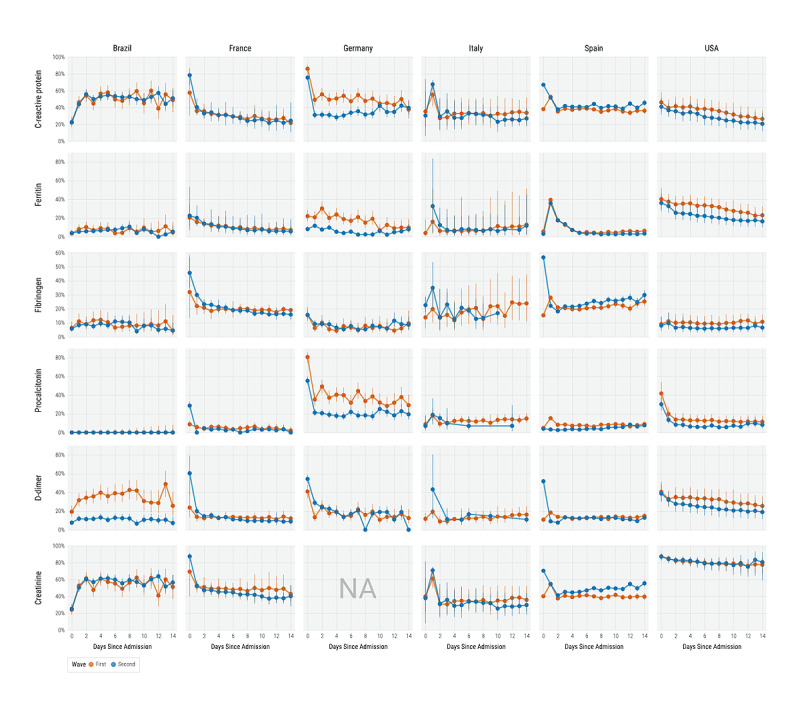
Laboratory testing rates across hospitalization days in each country. Error bars indicate 95% CIs. Laboratory data for creatinine were unavailable for health care systems in Germany. NA: Not Available.

When comparing the overall laboratory testing rates during hospitalization, as reported in Figure 5, we observed that overall testing rates among all patients for procalcitonin were significantly lower across hospitalization days in the second wave than in the first wave. Overall procalcitonin testing rates among patients with severe disease were similarly significantly lower across hospitalization days in the second wave than in the first wave. There were no other significant changes in overall laboratory testing rates. We report laboratory testing rates within each country in Figure 6; creatinine laboratory data were not available from participating health care systems in Germany. Laboratory testing rates among countries varied significantly between the first and second waves. European countries exhibited the most changes in testing rates at admission (day 0). In the second wave, there was a significant increase in the D-dimer testing rate in France, Germany, and Spain, and in the CRP, creatinine, and fibrinogen testing rates in Spain. In Germany, there were significant decreases in the testing rates for CRP, ferritin, and procalcitonin at admission. In Brazil, there was a significant decrease in the testing rate for D-dimer at admission. In contrast, the United States did not have any significant changes in laboratory testing rates at admission. In all countries, except Brazil and Spain, laboratory testing rates in the second wave were generally lower during the second week of hospitalization. In Spain, second hospital week testing rates for CRP, fibrinogen, and creatinine were higher in the second wave than in the first wave.

## Discussion

### Principal Findings

In this large EHR-based study, we employed a federated approach to rapidly aggregate and harmonize clinical data across 315 international hospitals from 6 countries that included 79,613 hospitalized patients with SARS-CoV-2 to offer insights on the evolving clinical trajectory of COVID-19 across the first and second waves. We found that patients hospitalized in the second wave were at significantly lower risk for severe COVID-19, corresponding to lower mean laboratory values for several inflammatory markers during the first week of hospitalization in the second wave than in the first wave.

In this study, we capitalized on the availability of real-world EHR data from participating international health care systems within the 4CE to capture pertinent clinical characteristics effectively and accurately. Despite the high heterogeneity in the health systems, we were able to rigorously perform quality checks across all health care centers using a multidisciplinary team approach that engages statisticians, informaticians, and clinicians. Additionally, the multinational nature of our data allowed us to identify country-level variations in temporal trends, as well as distinguish different clinical phenotypes and trajectories in the second wave compared with the first wave of the pandemic.

Notably, our federated approach demonstrated several advantages over methods where hospitals transfer patient-level data to a central repository [30]. By keeping data local within hospitals, we avoided privacy concerns and regulatory barriers that often delay multisite research studies. By comparing the results from different hospitals, rather than treating all the data as a single combined data set, we could identify outliers that suggested data quality problems. Finally, by having local data experts at each site running the database queries, we could leverage their help in addressing these data quality concerns [31,32]. Although more sophisticated analyses, such as machine learning models or robust multivariable models that can adjust for multiple sources of various types of bias, are harder to implement in a federated architecture than in a central repository, this study demonstrated that our federated approach enabled us to obtain early clinical insights into the evolving pandemic and helped us gain confidence in the data.

Our data demonstrated a near uniform peak in country-specific hospitalizations in the first wave and a variable peak in country-specific hospitalizations in the second wave, reflecting country-specific patterns in the resurgence of COVID-19–related hospitalizations that were consistent with international tracking sites [1,9,10]. In all countries, except Brazil and the United States, the second wave peak was characterized by a lower intensity compared to the first wave peak, possibly reflecting the effects of successful COVID-19 mitigation measures implemented after the first wave [33,34].

Consistent with previous single-country studies, we observed that patients hospitalized in the second wave had an overall lower risk for severe COVID-19 than patients hospitalized in the first wave [6,12,35,36]. On further stratifying our analyses by country, we were able to observe that patients in Spain instead had a significantly higher risk for severe disease in the second wave, contrary to what we observed in France and the United States. Although the reasons for the increased risk of severe COVID-19 during Spain’s second wave are unclear and likely complex, it is consistent with international tracking sites that indicated increased mortality rates and health care resource use in Spain’s second wave, and it reflects the importance of being able to identify country-specific variations in our data [37]. We further note that the data from Spain originated from 1 hospital and were likely subject to some forms of bias. Even when stratifying by demographic subgroups, we observed similar patterns indicating reduced risk for severe COVID-19 in the second wave, particularly among patients aged 26 to 49 and among black patients. However, while the risk of severe disease for the entire population was lower in the second wave for patients aged 26 to 49, country-specific results demonstrated possible collider bias and small-sample bias in that the individual estimates for Brazil, France, Germany, and Spain were not statistically significant and were imprecise with wide CIs. This is likely due in large part to the smaller sample size of this age group in each country and reflects the need for multicenter studies to improve power. While one might expect to see a less severe disease later in the course of the pandemic due to changes in patient populations over time, improved clinical care, and greater utilization of health care resources compared to the beginning of the pandemic, it is unclear why we did not observe similar relationships for other patient groups. These possible discrepancies are likely due to a variety of different factors, as noted above, as well as sources of bias in our data. Further investigation into these country-specific demographic differences in the change in severe disease risk over the course of the pandemic is warranted and is ongoing in the 4CE.

Our observations comparing laboratory values between the first and second waves support our finding that patients hospitalized during the second wave compared to the first wave had a lower risk for severe disease. We found that mean laboratory values in the second wave exhibited considerable improvement toward typical physiological values compared to the first wave, especially those of inflammatory markers. In particular, the mean values of the positive acute phase reactant CRP were lower across the first week of hospitalization in the second wave than in the first wave, while the mean values of the positive acute phase reactants ferritin, fibrinogen, and procalcitonin were lower at day 7 [38]. This indicates that, on average, patients hospitalized during the second wave may have had less overall systemic inflammation at admission and had improved inflammatory states during the first week of hospitalization in comparison to patients admitted in the first wave [39-41]. Considering that there were no new major effective pharmacologic therapies for patients with COVID-19 introduced between the first and second waves, these general patterns may be reflective of a less vulnerable patient population in the second wave, as well as improved general clinical management strategies of COVID-19 in the second wave [42-50]. Ongoing 4CE analyses are further investigating these findings.

We further observed variations in laboratory testing rates among countries between the first and second waves. These changes in laboratory testing rates at admission may be reflective of greater understanding of COVID-19 pathophysiology and clinical trajectories leading to changes in clinical protocols. For example, there was a significant increase in the testing rate of D-dimer at admission in France, Germany, and Spain in the second wave. This particular change in clinical practice may have been driven in part by the growing literature supporting the association of high D-dimer values with worse outcomes in COVID-19 and the possibility of using D-dimer to clinically classify and evaluate the prognosis of COVID-19 patients [51-55]. Further, although there were no significant changes in laboratory testing at admission in the United States, we observed that testing rates across hospitalization days were generally higher than in other European countries regardless of wave. Future investigations are warranted to infer why we observed these patterns.

### Study Limitations

We acknowledge several limitations for this EHR-based observational cohort study. This study was limited to patients who were admitted to a hospital, either because they experienced more severe illness or because they had other possibly biasing conditions; as with many EHR-based studies, we were unable to ascertain the precise reasons for admission. Similar to other EHR-based studies, we were not able to validate if patients were hospitalized due to COVID-19 or happened to have a positive test when admitted for an unrelated medical condition. Thus, we could not completely mitigate selection bias or misclassification bias in our cohort identification. Due to the limited scope of the extracted aggregate data, we could not effectively control for patient-level potentially confounding variables such as comorbidities, medication use (both prior to and during hospitalization), and other societal and environmental factors, all of which can induce many types of biases [56]. Data pertaining to certain countries, most notably Brazil and Germany, may have been subject to small sample bias. Furthermore, mean laboratory values at later days of hospitalization were subject to censoring (transfer, discharge, and death) and thus dropout bias, so we could not effectively compare mean laboratory values within a single wave at different timepoints. However, we believe that facilitating comparisons at identical timepoints between different waves is not subject to as much dropout bias. In an effort to provide information regarding the nature of censoring that existed in the data, we report in Multimedia Appendix 3 the proportion of patients who were alive and remained in the hospital across hospitalization days for each country by wave. Further, considering the aforementioned limitations, we took special caution to make conclusions that were mostly descriptive in nature. In the future, we hope to disaggregate EHR data to the patient level in order to adjust for many of these biases, if possible, under institutional review board approval.

### Conclusions

For assessing the evolving epidemiology, pathophysiology, and health care dynamics of the COVID-19 pandemic, we leveraged EHR data in a large international cohort of hospitalized patients with SARS-CoV-2 to rapidly characterize the clinical course of patients admitted to hospital during the first 2 major waves of the pandemic. We were able to characterize changes in hospitalization rates, demographic characteristics, severity risk, and mean laboratory values using data from 79,613 patients across 315 health care systems in 6 countries. Our study’s federated approach demonstrates the feasibility and power of leveraging real-world EHR data from multiple countries to support our understanding of evolving pandemics such as COVID-19.

## Appendix

Multimedia Appendix 1 Descriptions of CSV files generated at participating health care systems.

Multimedia Appendix 2 Prevalence of demographic subgroups in the first and second waves for the entire cohort and by country, and estimated absolute risk for severe COVID-19 by demographic subgroups inclusive of 5 Veterans Affairs health care systems. Error bars indicate 95% CIs. *Significant difference by nonoverlapping 95% CIs.

Multimedia Appendix 3 Proportion of patients who were alive and still in the hospital across hospitalization days.

## Abbreviations

4CE: Consortium for Clinical Characterization of COVID-19 by EHR
CRP: C-reactive protein
EHR: electronic health record
ICD: International Classification of Diseases
RR: relative risk
VA: Veterans Affairs
