# Authorship Correction: International Changes in COVID-19 Clinical Trajectories Across 315 Hospitals and 6 Countries: Retrospective Cohort Study

**DOI:** 10.2196/34625

**Published:** 2021-11-30

**Authors:** Griffin M Weber, Harrison G Zhang, Sehi L'Yi, Clara-Lea Bonzel, Chuan Hong, Paul Avillach, Alba Gutiérrez-Sacristán, Nathan P Palmer, Amelia Li Min Tan, Xuan Wang, William Yuan, Nils Gehlenborg, Anna Alloni, Danilo F Amendola, Antonio Bellasi, Riccardo Bellazzi, Michele Beraghi, Mauro Bucalo, Luca Chiovato, Kelly Cho, Arianna Dagliati, Hossein Estiri, Robert W Follett, Noelia García Barrio, David A Hanauer, Darren W Henderson, Yuk-Lam Ho, John H Holmes, Meghan R Hutch, Ramakanth Kavuluru, Katie Kirchoff, Jeffrey G Klann, Ashok K Krishnamurthy, Trang T Le, Molei Liu, Ne Hooi Will Loh, Sara Lozano-Zahonero, Yuan Luo, Sarah Maidlow, Adeline Makoudjou, Alberto Malovini, Marcelo Roberto Martins, Bertrand Moal, Michele Morris, Danielle L Mowery, Shawn N Murphy, Antoine Neuraz, Kee Yuan Ngiam, Marina P Okoshi, Gilbert S Omenn, Lav P Patel, Miguel Pedrera Jiménez, Robson A Prudente, Malarkodi Jebathilagam Samayamuthu, Fernando J Sanz Vidorreta, Emily R Schriver, Petra Schubert, Pablo Serrano Balazote, Byorn WL Tan, Suzana E Tanni, Valentina Tibollo, Shyam Visweswaran, Kavishwar B Wagholikar, Zongqi Xia, Daniela Zöller, The Consortium for Clinical Characterization of COVID-19 by EHR (4CE), Isaac S Kohane, Tianxi Cai, Andrew M South, Gabriel A Brat

**Affiliations:** 1 Department of Biomedical Informatics Harvard Medical School Boston, MA United States; 2 BIOMERIS (BIOMedical Research Informatics Solutions) Pavia Italy; 3 Clinical Research Unit Botucatu Medical School São Paulo State University Botucatu Brazil; 4 Division of Nephrology Department of Medicine Ente Ospedaliero Cantonale Lugano Switzerland; 5 Department of Electrical, Computer and Biomedical Engineering University of Pavia Pavia Italy; 6 Information Technology Department Azienda Socio-Sanitaria Territoriale di Pavia Pavia Italy; 7 Unit of Internal Medicine and Endocrinology Istituti Clinici Scientifici Maugeri SpA SB IRCCS Pavia Italy; 8 Massachusetts Veterans Epidemiology Research and Information Center Veterans Affairs Boston Healthcare System Boston, MA United States; 9 Department of Electrical Computer and Biomedical Engineering University of Pavia Pavia Italy; 10 Department of Medicine Massachusetts General Hospital Boston, MA United States; 11 Department of Medicine David Geffen School of Medicine University of California, Los Angeles Los Angeles, CA United States; 12 Health Informatics Hospital Universitario 12 de Octubre Madrid Spain; 13 Department of Learning Health Sciences University of Michigan Medical School Ann Arbor, MI United States; 14 Department of Biomedical Informatics University of Kentucky Lexington, KY United States; 15 Department of Biostatistics, Epidemiology, and Informatics University of Pennsylvania Perelman School of Medicine Philadelphia, PA United States; 16 Institute for Biomedical Informatics University of Pennsylvania Perelman School of Medicine Philadelphia, PA United States; 17 Department of Preventive Medicine Northwestern University Chicago, IL United States; 18 Institute for Biomedical Informatics University of Kentucky Lexington, KY United States; 19 Medical University of South Carolina Charleston, SC United States; 20 Department of Computer Science Renaissance Computing Institute University of North Carolina at Chapel Hill Chapel Hill, NC United States; 21 Department of Biostatistics Harvard T.H. Chan School of Public Health Boston, MA United States; 22 Department of Anaesthesia National University Health System Singapore Singapore; 23 Institute of Medical Biometry and Statistics Faculty of Medicine and Medical Center University of Freiburg Freiburg Germany; 24 Michigan Institute for Clinical & Health Research Informatics University of Michigan Ann Arbor, MI United States; 25 Laboratory of Informatics and Systems Engineering for Clinical Research Istituti Clinici Scientifici Maugeri SpA SB IRCCS Pavia Italy; 26 Clinical Hospital of Botucatu Medical School São Paulo State University Botucatu Brazil; 27 Informatique et archivistique médicales unit Bordeaux University Hospital Bordeaux France; 28 Department of Biomedical Informatics University of Pittsburgh Pittsburgh, PA United States; 29 Department of Neurology Massachusetts General Hospital Boston, MA United States; 30 Department of Biomedical Informatics Hôpital Necker-Enfants Malade, Assistance Publique Hôpitaux de Paris University of Paris Paris France; 31 Department of Biomedical Informatics, Institute for Digital Medicine National University Health System Singapore Singapore; 32 Internal Medicine Department Botucatu Medical School São Paulo State University Botucatu Brazil; 33 Department of Computational Medicine & Bioinformatics, Internal Medicine, Human Genetics, and Public Health University of Michigan Ann Arbor, MI United States; 34 Division of Medical Informatics Department of Internal Medicine University of Kansas Medical Center Kansas City, KS United States; 35 Data Analytics Center University of Pennsylvania Health System Philadelphia, PA United States; 36 Department of Medicine National University Health System Singapore Singapore; 37 Department of Neurology University of Pittsburgh Pittsburgh, PA United States; 38 Section of Nephrology Department of Pediatrics Brenner Children's Hospital, Wake Forest School of Medicine Winston Salem, NC United States; 39 see Authors’ Contributions

In “International Changes in COVID-19 Clinical Trajectories Across 315 Hospitals and 6 Countries: Retrospective Cohort Study” (J Med Internet Res 2021 Oct 11;23(10):e31400), two errors were noted.

In the originally published paper, equal contribution of the last three authors was not noted. This has been corrected to add a note of equal contribution to the last three authors, as well as to add a statement to the Authors' Contributions section denoting the nature of equal contributions.

In the originally published paper, an equal contribution footnote was applied to authors Griffin M Weber, Harrison G Zhang, and Sehi L'Yi.

In the corrected paper, the equal contribution footnote has been applied to authors Griffin M Weber, Harrison G Zhang, Sehi L'Yi, Tianxi Cai, Andrew M South, and Gabriel A Brat.

The Authors’ Contribution section has been updated to include the following statement:

These authors contributed equally: GMW, HGZ, SL. These authors jointly supervised the work: TC, AMS, GAB.

The correction will appear in the online version of the paper on the JMIR Publications website on November 30, 2021, together with the publication of this correction notice.

