# Supplementary material for: International Changes in COVID-19 Clinical Trajectories Across 315 Hospitals and 6 Countries: Retrospective Cohort Study
Source: J Med Internet Res. 2021 Oct 11;23(10):e31400. doi: 10.2196/31400 (PMC8510151; doi:10.2196/31400)
Supplement: Multimedia Appendix 1 [file jmir_v23i10e31400_app1.docx]

**Multimedia Appendix 1.** Descriptions of CSV files generated at participating health care systems.

| DailyCounts.csv | The file contains one row per calendar date. The columns include for each date the cumulative number of patients admitted, the cumulative number of patients who had developed severe disease, cumulative number of patients who have died, and the number of patients in hospital and the number of patients who became severe and are in hospital. |
| --- | --- |
| ClinicalCourse.csv | The file contains one row per day since admission, containing aggregate data. The columns include the number of days since admission (where day = 0 is admission date). the number of patients in hospital, and the number of patients who became severe and are in hospital. Patients who were discharged and then readmitted were counted for each day they were an inpatient. |
| Demographics.csv | The file contains breakdown counts for the total cohort and the severe cohort by sex, age group (0-25, 26-49, 50-69, 70-79, and 80+ years old), as well as race and ethnicity category (Black, White, and other). Reporting of the race categories was restricted to the United States sites. |
| Labs.csv | The file contains one row per day since admission and laboratory test, identified using LOINC code. The columns include, for both total cohort and severe cohort, the number of patients who had a test result on that day, the mean and standard deviation of the test result, and the mean and standard deviation of the natural logarithm of the test result plus 0.5. If a patient had multiple results for the same test on the same day, those were replaced by the mean of that day’s test results before averaging across all patients. |
