# Supplementary figures and images for: International Changes in COVID-19 Clinical Trajectories Across 315 Hospitals and 6 Countries: Retrospective Cohort Study
Source: J Med Internet Res. 2021 Oct 11;23(10):e31400. doi: 10.2196/31400 (PMC8510151; doi:10.2196/31400)

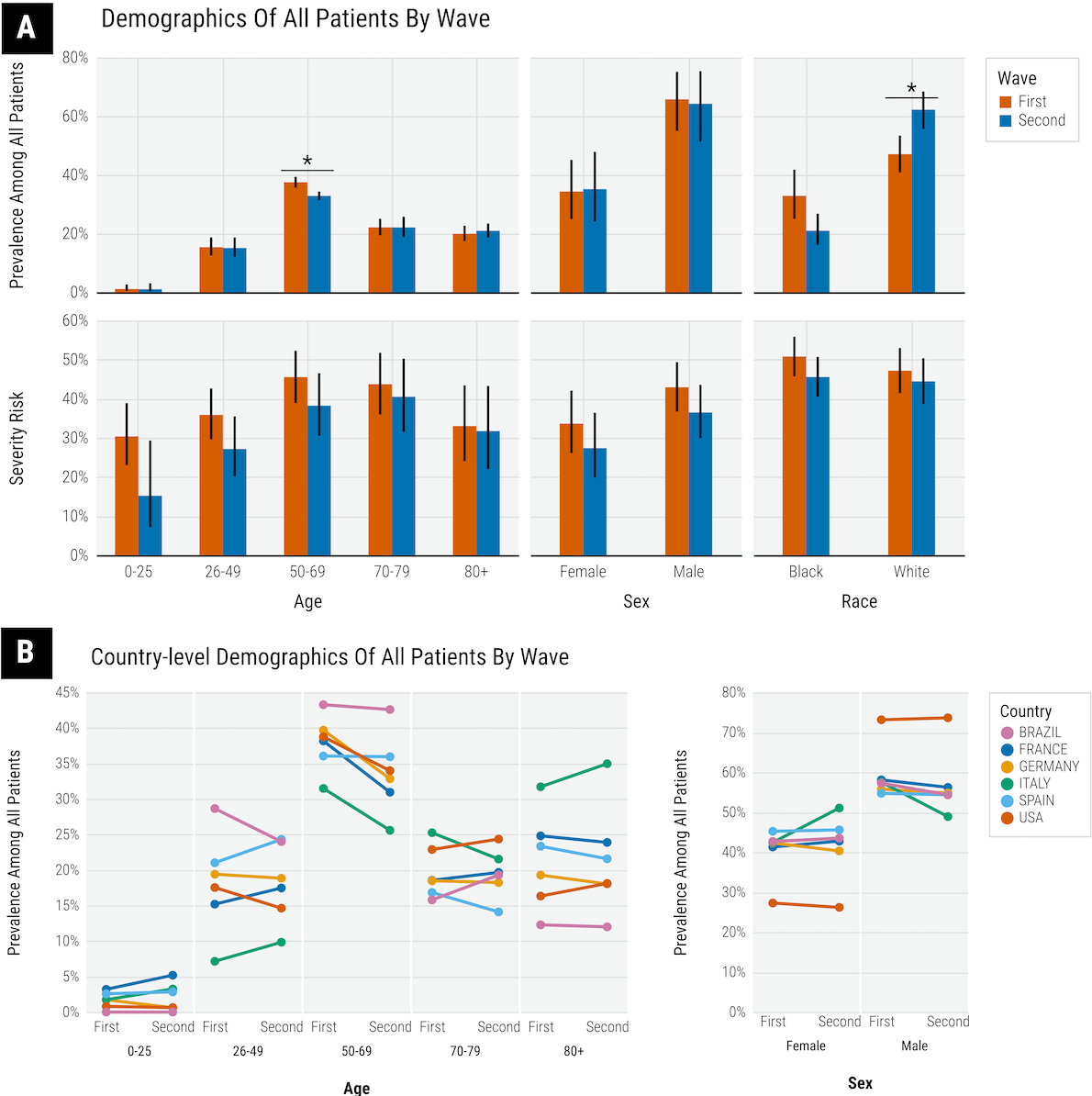

Supplement: Multimedia Appendix 2 [file jmir_v23i10e31400_app2.png]

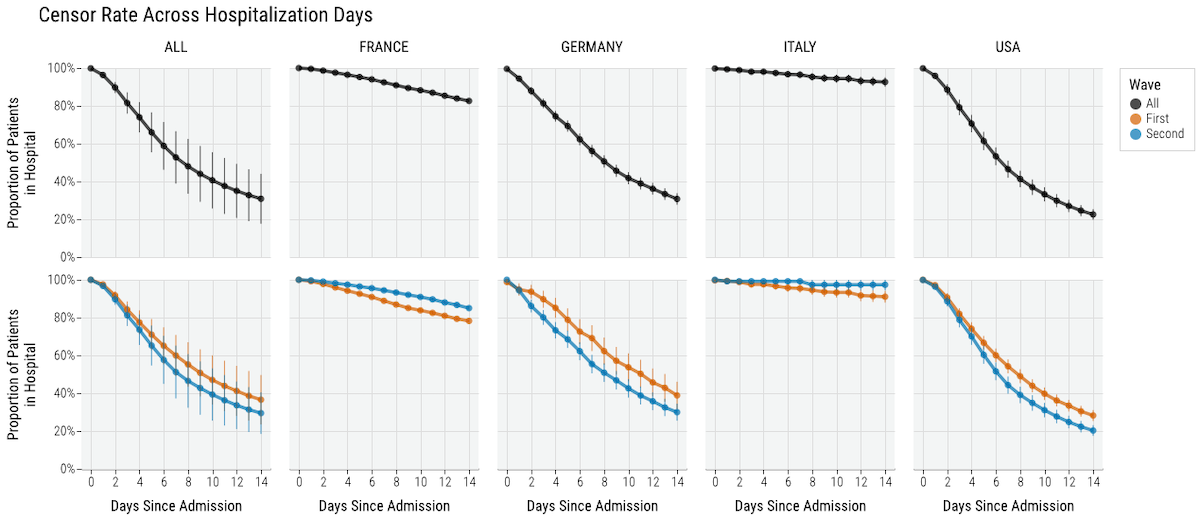

Supplement: Multimedia Appendix 3 [file jmir_v23i10e31400_app3.png]
